# Sweet Wine Production from the Side-Stream of Industrial Corinthian Currant Processing: Product Quality, Antioxidant Capacity, and Volatilome

**DOI:** 10.3390/molecules28145458

**Published:** 2023-07-17

**Authors:** Iris Plioni, Eleni Michalopoulou, Athanasios Mallouchos, Stavros Plessas, Gerasimos Gotis, Argyro Bekatorou

**Affiliations:** 1 Department of Chemistry, University of Patras, 26504 Patras, Greece; plioni@upatras.gr (I.P.); elenimic66@gmail.com (E.M.); 2 Department of Food Science and Human Nutrition, Agricultural University of Athens, 75 Iera Odos, 11855 Athens, Greece; amallouchos@aua.gr; 3 Laboratory of Food Processing, Department of Agriculture Development, Democritus University of Thrace, 68200 Orestiada, Greece; splessas@agro.duth.gr; 4 Department of Chemical Engineering, University of Patras, 26500 Patras, Greece; gerasimosgotis@gmail.com

**Keywords:** antioxidant capacity, Corinthian currants, fortification, phenolics, raisins, side-stream, sweet wine, volatilome

## Abstract

In the frame of efforts to add value to the Mediterranean currant cultivation and processing sectors, which is essential for their sustainability, sweet wine production is proposed from the finishing side-stream (FSS) of premium quality Corinthian currants, involving complete fermentation using an alcohol-tolerant yeast followed by (i) the addition of FSS to extract sugars or (ii) syrup made from FSS to adjust sweetness. Wine was also made by (iii) ceasing fermentation at the desired sugar level by ethanol addition. The non-fortified wines had 15.2–15.5% ethanol, 115–145 g/L residual sugar, 7.2–7.6 g/L titratable acidity, low volatile acidity (VA; <0.33 g/L), 280–330 mg/L phenolic content (TPC) (as gallic acid), and 23.8–35.6 mg/L antioxidant capacity (AC) (as ascorbic acid). In total, 160 volatiles were identified by SPME GC-MS, including compounds derived from the grapes, the raisin drying, and the fermentation process. The non-fortified wines had better characteristics (mainly VA, AC, and TPC) than the fortified wine, while sweetness adjustment by FSS is the simplest and lowest cost method since it does not involve ethanol or syrup addition. The proposed methods can lead to good quality sweet wines with a characteristic fruity (grape/raisin) flavor that could be commercialized as specialty raisin beverages or liqueurs.

## 1. Introduction

Corinthian currants (small black raisins produced by natural sun drying of *Vitis vinifera* L. var. Apyrena grapes), are a historic Greek product cultivated since antiquity. During the second half of the 19th century and beyond, their cultivation and trade were significant factors that drove the economic growth of the country [1,2]. The currants’ production does not involve heat treatments, apart from natural sun or shade drying, nor the addition of additives, except for coating with an edible oil upon customer request. They can be consumed as a snack, added to bakery and confectionery products, and used in cooking, or they can be processed into syrups and concentrates, for use as sweeteners, alcohol, vinegar, and beverages [2,3,4]. Today, Corinthian currants are still one of the main exporting commodities of the country; the highest quality currant being Vostitsa (Protected Designation of Origin), a highly nutritious product, rich in antioxidant phenolics and micronutrients [5,6,7]. However, the currant sector is shrinking due to challenges such as high production costs, difficult cultivation practices, and extraordinary circumstances such as the economic crisis, the COVID-19 pandemic, and adverse weather conditions. In order to ensure the sector’s sustainability, governmental subsidies are inevitably required, as well as the adaptation of strategies for new product development, waste minimization, and by-product utilization to create added value [1,2,3,4,8,9]. 

The main by-product of Corinthian currant processing is the industrial finishing side-stream (FSS), i.e., the substandard currants (mainly due to size) that are separated from the main bulk of the marketable product. The FSS generated from a Corinthian currant processing company accounts for about 5–6% of the total processed currants, and has great potential for exploitation through the production of a variety of added value products. It contains ~70% fermentable sugar, it has a rich volatilome, including compounds produced by the sun-drying process, and it has increased antioxidant capacity (AC), lipid content, and total phenolic content (TPC) compared to the marketable product [2]. FSS, aiming to upgrade it within the biorefinery concept, has recently been proposed as raw material for the production of dry wine [2], vinegar [3], syrup [4], and bacterial cellulose (as a potential prebiotic additive or packaging material) [8]. The production of added value products from FSS can increase the revenue and sustainability of the currant processing sector, and support the local economies and the local biodiversity. These works also proposed the development of novel production methods that could lead to products with sensory appeal, nutritional quality, increased shelf-life, and a low production cost, which are fundamental requirements for industrial application [10,11].

Likewise, emerging technologies in winemaking also aim to reduce production times and costs, and provide a higher nutritional value through the efficient extraction of bioactive components, an extended shelf-life, lower sulfite addition, etc. [11]. Generally, fermentation of sugar-rich, food-grade wastes and by-products for alcoholic beverages and distillates’ production, is a strategy that can lead to significant added value for the agri-industrial sectors [12]. 

Sweet wines include special types such as botrytized wines, wines made by dried grapes or heat-concentrated grape juice, icewines, and a variety of white, rosé, and red wines that are produced worldwide. Sweet, non-botrytized wine production includes (a) the addition of unfermented grape juice to a dry wine to adjust sweetness, or (b) the addition of a distillate (fortifying wine spirit or even rectified alcohol) to a partially fermented wine in order to cease the fermentation at the desired sweetness. Additional fortification by potable alcohol addition may be performed to adjust the wine strength to the desired ethanol concentration, which may be up to 19–21% [13]. Challenges in sweet wine production are related to the maceration, vinification, and ageing processes, the high cost of the added alcohol, the climate conditions, the involved microorganisms (osmotolerance, alcohol tolerance), etc. [11,13,14,15,16]. Fortification with alcohol to cease the fermentation and obtain a sweet wine, may lead to wines that lack the typical fermentation aromas and have imbalanced acidity (low) or sweetness (high). Therefore, selected osmotolerant yeasts have been proposed for sweet wine making, including wines from raisin extracts [14,15]. 

Wines from raisins are amber (yellow–brown) rather than red in color [2,17], mainly due to the sun drying, which alters the color derived from phenolic compounds. As discussed previously [2,18], raisins, unlike overripe grapes, are dehydrated to a level that does not allow crushing to extract the juice or spontaneous fermentation. Therefore, raisins are not intended for conventional wine production, and wine made by aqueous raisin extract should be properly labelled as raisin beverage or liqueur [18]. 

**Figure 1:**
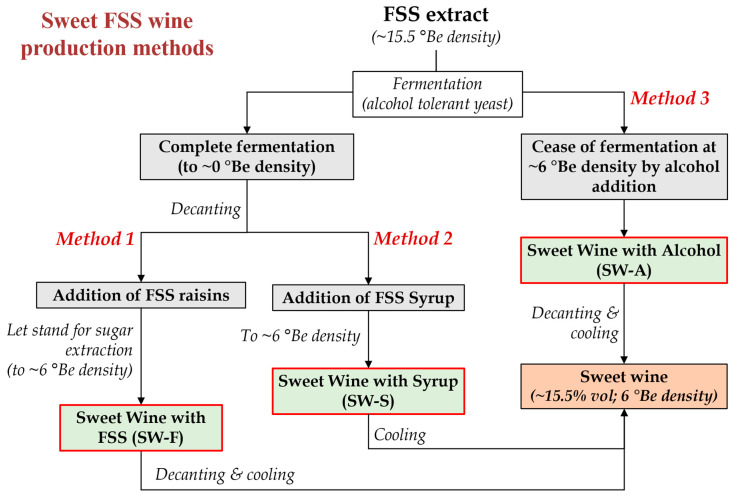
Methods applied for the production of sweet wines from Corinthian currants finishing side-stream (FSS) (°Be: Baumé scale hydrometer density).

Considering the above, in this study, three different methods are proposed and compared for the production of sweet wines from FSS extracts of the premium quality Vostitsa currants (Figure 1), involving: wine production by complete fermentation with an alcohol tolerant yeast, followed by the addition of whole FSS raisins to extract their sugars in the wine (Method 1; SW-F wine), or the addition of syrup made from FSS (Method 2; SW-S wine) to obtain the desired sweetness, or ceasing fermentation at the desired sugar level by potable alcohol addition (Method 3; SW-A wine). The chemical composition, volatilome, antioxidant and phenolic content, and sensory properties of the produced sweet wines were evaluated and compared.

## 2. Results

### 2.1. Composition of the Sweet Wines Made from FSS

**Figure 2:**
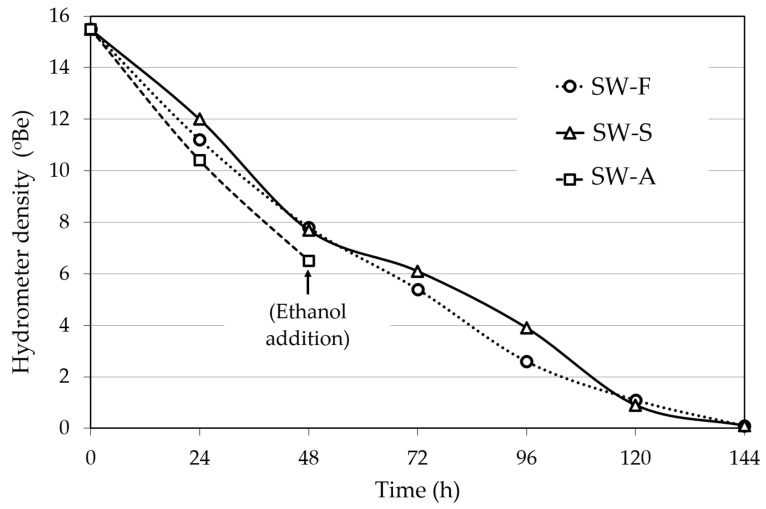
Course of fermentation of Corinthian currants finishing side-stream (FSS) extracts for sweet wine production. SW-F: Sweet wine with added FSS raisins to adjust sweetness. SW-S: Sweet wine with added FSS syrup to adjust sweetness. SW-A: Sweet wine with alcohol added to cease fermentation at the desired sweetness level.

The fermentation kinetics of the FSS extracts (density of the fermenting liquid versus time) of the three different sweet wine methods are presented in Figure 2. In Methods 1 and 2, the fermentation was complete, and all sugar was utilized at about 144 h, yielding alcohol contents (av.; % vol.) of 15.3% for SW-F wine and 15.0% for SW-S wine (Table 1). The average alcohol level in the fortified SW-A wine was 16.0% (Method 3; Table 1). 

Methanol was not detected in any of the wines, as expected since it was not present in the raw material [2]. 

The sugar levels in the wines differed (*p* < 0.05), and were in the average ranges of 44–68 g/L glucose, 63–71 g/L fructose, 111–139 g/L total sugar, and traces of sucrose in all cases (Table 1). 

The total titratable acidity (TTA) (av. 7.4 g/L, as tartaric acid) and pH values (av. 3.9) of SW-F and SW-S wines were similar (*p* < 0.05) (Table 1), while SW-A wine had a slightly lower TTA (6.4 g/L) and pH (3.83).

The volatile acidity (VA) of the wines was statistically different (*p* < 005) and in the average range of 0.3–0.7 g/L (as acetic acid), with the highest value being that of SW-A (Table 1). 

Regarding the analysis of individual organic acids by HPLC, the main organic acids were tartaric (2.0–2.6 g/L), malic (3.3–3.6 g/L), and succinic acid (2.0–2.8 g/L), while all wines contained citric and acetic acid at lower concentrations (<1.0 g/L) (Table 1).

Statistically significant differences were also observed among the three types of wine regarding the TPC (Folin–Ciocalteu reaction), AC (radical scavenging), and polyphenol content (PPC) (ferric ammonium citrate reaction). Specifically, the TPC was in the (av.) range 244–325 g GA/L, AC was 18–35 mg AA/L, and PPC was 459–576 mg/L; the highest values were observed in the case of SW-F wine. 

Finally, free and total sulfite, which was analyzed in order to regulate its residual content in the final products, was found at very low levels (<40 mg/L). Sulfite was added (as potassium metabisulfite) during the production of the wines, as described in the Section 4, and is common practice in winemaking to avoid spoilage, avoid spontaneous fermentation, and act as an antioxidant, as well as for its ability to bleach pigments and suppress oxidized odors [13].

### 2.2. Volatilome of the Sweet Wines

An analysis of the headspace, aroma profile of the sweet wines was carried out by solid phase micro-extraction, gas chromatography–mass spectrometry (SPME GC-MS) identifying 160 compounds (43 esters, 6 lactones, 31 alcohols, 12 organic acids, 32 carbonyl compounds, 19 terpenes, 11 hydrocarbons, and 6 other compounds) (Table 2). The origin of these compounds (presence in wines, raisins, grapes, etc.) and their effect on food aroma has been described in detail in [2]. These compounds were identified in all sweet wines, with quantitative differences (expressed as % normalized peak areas as retrieved by the semi-quantitative GC-MS analysis).

The main fruity esters identified in the wines were isoamyl acetate (banana), ethyl hexanoate (pineapple, banana), ethyl octanoate (banana, pear, winey, brandy), and ethyl acetate (ethereal, grape, rummy), which were found in all wines at levels of 5–24%. All other esters were found at levels below 1%, except ethyl decanoate (fruity, apple, grape, brandy), which was 3.5–6% in SW-S and SW-A.

The main alcohol that was found in sweet wines at high levels (17–37%) compared to other compounds, was the fermentation product isoamyl alcohol (fruity, banana, whiskey, cognac); the highest levels (>36%) were of those found in SW-F and SW-S. Another alcohol found at considerable high levels (6–10%) was 2-phenylethanol (floral, rose, honey; product of both grape and yeast metabolism) [2], again prevailing in SW-F and SW-S wines. All other alcohols were found at levels below 0.5%.

Volatile organic acids were found at low concentrations (<0.2%), expect acetic acid (0.3–0.8%; higher level in SW-S), and octanoic acid (0.5–1.2%). Decanoic acid was found at considerable levels in SW-S and SW-A wines (0.3–0.6%) compared to SW-F (0.05%). 

The main carbonyl compounds identified were acetaldehyde (0.2–0.3%; aldehydic, fruity), hexanal (0.1–0.7%; aldehydic, grassy, fruity), octanal (0.1–0.5%; green, fatty), furfural (0.1–0.5%; nutty, almond, caramellic), and benzaldehyde (0.0–0.7%; cherry, nutty, almond). All these compounds were found at higher levels in the headspace of wines SW-F and SW-S, which on total contained 3.6% and 2.7% carbonyl compounds, respectively, compared to only 0.8% in SW-A. It is obvious that the increased carbonyls in SW-F are due to their extraction from FSS, which was added, post-fermentation, to increase the sweetness of the wine.

The total amount of terpenes in the headspace of all wines was 0.2–0.3%, with most found at levels < 0.01%. Important aroma terpenes, identified at levels > 0.01%, were D-limonene (citrus), β-citronellol (floral, rose, citrus), β-damascenone (fruity, rose, plum), linalool (citrus, floral, green), and trans-geranylacetone (fruity, tropical, floral).

Of the six lactones that were identified, three were found at levels > 0.01%, i.e., γ-butyrolactone (caramel, milky, peach), δ-octalactone (coconut, tropical, dairy), and γ-nonalactone (coconut, buttery, milky). Most lactones have previously been found in grapes, raisins, or wine, except 2-hexen-1,4-lactone, which has not previously been reported in grapes or wines [2].

Various other compounds, deriving from the raw material or the raisin-drying process, such as dimethyl sulfide, acetal, various acetyl furans, and methyl eugenol, were identified at levels < 0.02%, except acetal (ethereal, nutty, earthy), which was found at ~0.25% in all samples.

Finally, several alkanes and alkenes were identified, some of which have not previously been identified in grapes, raisins, or wine (C7–10, C12 alkanes, 1/2-octenes), except for dry wine from FSS [2], and may be a result of microbial spoilage of the FSS during its generation and handling in the factory. 

### 2.3. Sensory Properties of the Sweet Wines

**Figure 3:**
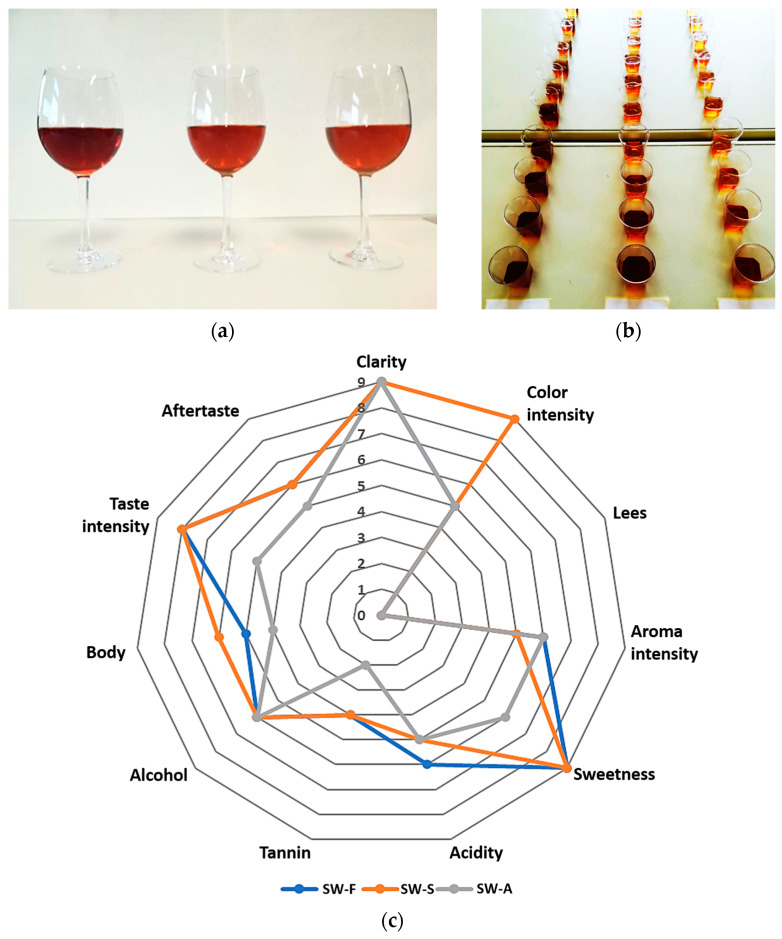
(**a**) Sweet wines produced from Corinthian currants finishing side-stream (FSS). From left to right: SW-S: Sweet wine with added FSS syrup to adjust sweetness. SW-F: Sweet wine with added FSS raisins to adjust sweetness. SW-A: Sweet wine with alcohol added to cease fermentation at the desired sweetness level. (**b**) Samples for sensory evaluation. (**c**) Spider web diagrams obtained from the descriptive sensory evaluation scores for visual, olfactory, and gustative descriptors of the sweet wines.

The results of the sensory evaluation are presented in Table 3 and Figure 3. Regarding their appearance, all three wines were described as clear, and with no observable sediment. The color was described as brown amber for all wines, with medium intensity for SW-F and SW-A, and deeper for SW-S wine (due to the addition of brown raisin syrup) (Figure 3a).

Regarding the aroma evaluation of all wines, it was described as fruity, characteristic of raisins and grapes, with a medium intensity and slight variations between samples (less intense in the case of SW-S wine).

Some differences were also noted by the evaluators regarding the taste of the sweet wines. Specifically, the taste of all wines was described as sweet and fruity (currants, raisins), while SW-F and SW-S wines were described as sweeter and fruitier compared to SW-A, which was described as weaker regarding all of its gustatory descriptors, except alcohol (slightly stronger). SW-F was described as slightly more acidic in taste, and SW-S as richer in body.

## 3. Discussion

As described in the Introduction section, the production of sweet wines may face challenges such as the high cost of the alcohol added for their fortification or the need for osmotolerant and alcohol tolerant yeasts [11,13,14,15,16]. Fortification with alcohol may increase the production cost of a sweet wine vertically and may lead to wines being deprived of typical fermentation aromas and with imbalanced acidity (usually low) or sweetness (high) [13]. In this study, in the frame of efforts to develop added value products by the exploitation of FSS, three methods for sweet wine making are proposed and compared (Figure 1). The starting raw material was aqueous FSS extract of high sugar content (~264 g/L), and the fermentation took place by the alcohol tolerant strain *Saccharomyces cerevisiae* AXAZ-1 [2]. The method relied on the complete conversion of sugars to produce at least 15% *v*/*v* ethanol. In the dry wine that was produced, an appropriate amount of either FSS (SW-F wine) or brown syrup made from FSS (SW-S wine) were added to adjust the sweetness, post-fermentation. Therefore, these methods do not require the addition of alcohol, which would greatly increase the cost of production due to the high taxation of ethanol. Wine was also made by fortification with alcohol for comparison (SW-A wine). The lower–upper limits of the various oenological parameters of the produced wines are discussed in this section, while all analyses took place right after the wines’ production (no storage or stabilization treatment applied).

The concentration of ethanol in the non-fortified wines (SW-F and SW-S), obtained by complete fermentation, was in the range 15.2–15.5% (Table 1), and their residual sugar contents were in the range 115–145 g/L, which are typical levels for many sweet fortified wines produced commercially or at research level [13,14,19]. However, the level of sugar may be adjusted if a different protocol is followed, e.g., different amount and contact time of the added FSS to extract sugars in the wine or a different amount of added FSS syrup.

The VA of the non-fortified wines was low (0.24–0.33 g/L) (Table 1), and lower than that of the SW-A wine (0.6–0.8 g/L), but all were within acceptable limits, i.e., below 1.5–2 g/L (depending on the type of sweet wine; ideally, acetic acid should not exceed 0.7 g/L in wines) [13,19], and similar to that reported for commercial sweet wines. For example, [19] reported VA levels (av.) of 0.6–1.2 g/L for natural sweet wines obtained by different grape dehydration processes, [20] reported levels of 0.8–1.0 g/L in sweet wine exposed to different storage conditions, and [14] reported levels of 1.2–1.4 g/L in fortified raisin wines. In [2], VA levels of 0.4–0.6 g/L were reported in dry wines made from the same batch of FSS as in this study. It should be noted that FSS was also found to have a VA of 2.0 g/kg [2], which was considered indicative of microbial spoilage, and therefore, better handling of this raw material in the factory was recommended if it is destined for high-quality, added value products such as wines, syrups, vinegars, etc. [2,3,4].

The non-fortified wines had TTA levels in the range 7.2–7.6 g/L (SW-A wine had 6.1–6.6 g/L) (Table 1), which are within the range reported for commercial sweet wines [13]. The higher acidity of the SW-F and SW-S wines is obviously due to the addition of FSS or FSS syrup, which increased the organic acid content of the wines post-fermentation. Additionally, the TTA of the FSS extracts had been adjusted before the fermentation by the addition of tartaric acid, as described in the Section 4, contributing to the final TTA of the wines. Acidity adjustment is important for both balancing the taste of the wines as well as for better resistance to spoilage [13], and is usually performed in the must to achieve a TTA level of 5.5–8.5 g/L (red wines being more appreciated at the lower end). However, the pH levels of all three wines are quite high for the given TTAs; therefore, this is an observation that should be further investigated, as a pH above 3.8 may provide a flat taste to the wine [13]. Other authors reported TTA levels (av.) of 4.6–9.1 g/L [19], 5.2–5.4 g/L [20], and 3.3–4.5 g/L [14], while dry wines made from the same batch of FSS [2] and the same yeast (free or immobilized) had TTA levels in the range 3.8–4.3 g/L (after stabilization treatments).

Among the wines, SW-A contained the lowest quantities of specific organic acids, as analyzed by HPLC (Table 1). Moreover, high levels of succinic (1.9–2.9 g/L) and malic acid (3.2–3.7 g/L) were found in all three wines, as previously reported [2] for dry FSS wine made by a more dilute FSS extract (11.3 °Be) (0.3–1.6 g/L malic acid, and 0.5–2.0 g/L succinic acid, after the post-fermentation treatments). Although the differences in organic acid concentrations among the samples were significant, they were at levels commonly found in wines. For example, [14] reported succinic acid levels (av.) of 0.2–0.3 g/L in sweet raisin wines produced by two osmotolerant strains in free and immobilized form.

The TPC of the sweet wines was at levels usually found in white and rosé wines [21]. Red sweet wines usually contain a much higher phenolic content (e.g., 894–3241 mg GAE/L [22] due to their production method (fermentation in the presence of grape marc). The SW-F and SW-S wines contained higher a TPC (in the range 280–330 mg GA/L) than SW-A (2.39–2.49 mg/L) (Table 1), obviously due to the addition of FSS or FSS syrup. Specifically, syrups made from the same FSS batch, condensed at a density of 38–40 °Be (65.4–69.4% sugar), had TPC levels (av.) of 1340–2133 mg/L [4], while FSS itself has 4760 mg/kg, as determined in its aqueous extract by the Folin–Ciocalteu reaction [2]. It should also be noted that the Corinthian currants are dark, almost black, grapes, but the produced wines, as in the case of most dried grape wines, are not red in color but have more yellow–brown (amber) shades [2,17] due to their dehydration process that affects the color imparted by the polyphenols of the skins (browning reactions).

The wines were additionally analyzed for polyphenol content (PPC) by a method based on the reduction of Fe(III). The PPC in the non-fortified wines was found to be 550–581 mg/L, also higher than that of SW-A (454–464 mg/L) (Table 1). The AC of the non-fortified wines SW-F and SW-F (23.8–35.6 mg AA/L) was also higher than that of SW-A (17.6–18.4 mg AA/L) (Table 1), which is consistent with their higher TPC of PPC. Dry wines previously produced by the same batch of FSS and yeast strain had AC levels (av.) of 21.0–26.3 mg AA/L [2]. In all cases, the non-fortified wines had better characteristics in terms of their antioxidant and phenolic contents. Therefore, the wine production method has a significant impact on these nutritionally important characteristics.

Regarding the volatilome of the wines, it can be observed that higher amounts of esters were contained in SW-A (72.1%), compared to SW-F (45%) and SW-S (44%) (Table 2), indicating that they were contained in the alcohol used for the fortification of this wine. Higher amounts of alcohols (45–51%) were contained in SW-S or SW-F, also indicating an effect of the complete fermentation process applied in the production of these wines. The total volatile organic acid levels were similar in samples SW-S or SW-A (2.1% and 2.3%, respectively), with lower levels found in SW-F (1.3%). Higher amounts of carbonyl compounds were contained in the non-fortified wines (3.6% in SW-F and 2.7% in SW-S), compared to SW-A (0.8%). Lactones were found in the wines at levels 0.1–0.16% (upper limit in SW-S), while hydrocarbons were found at very low amounts in all sweet wines (<0.04%). Finally, terpenes, which mainly derive from FSS and may be affected by the fermentation process (e.g., hydrolysis of glucosides and release of the aglycone forms), were at levels of 0.21–0.29% (upper limit in SW-F).

Based on the sensory, consumer-oriented evaluation (Table 2, Figure 3), the testers expressed a higher preference for the SW-F and SW-S wines; however, all testers stated that all three wines are pleasant to consume and can be commercialized as special-type Corinthian currant liqueurs.

## 4. Materials and Methods

### 4.1. Chemicals

The chemicals used in this study for the production and treatment of wines, and the methods of analysis, were: Std 0.1 M sodium hydroxide (NaOH) solution, ammonium sulfate [(NH_4_)_2_SO_4_], magnesium sulfate heptahydrate (MgSO_4_·7H_2_O), phenolphthalein, and fructose (Chem-Lab, Zedelgem, Belgium). Glucose, carboxymethyl cellulose (CMC), methanol, and ethanol (Fisher Scientific, Loughborough, UK). Saccharose (Chembiotin, Athens, Greece). Yeast extract and 2,2-diphenyl-1-picrylhydrazyl radical (DPPH) (Duchefa Biochemie, Haarlem, Netherlands). Glucose monohydrate for microbiology, potassium hydroxide (KOH), ammonia (NH_3_), agar, potassium dihydrogenphosphate (KH_2_PO_4_), 2-propanol, tartaric acid, and succinic acid (Merck, Germany). EDTA, sulfuric acid (H_2_SO_4_), malic acid, and gallic acid (Sigma-Aldrich, St. Louis, MO, USA). Acetic acid and hydrogen peroxide (H_2_O_2_) 30% (Carlo-Erba, Val de Reuil, France). Folin–Ciocalteu reagent (Scharlab S.L., Barcelona, Spain). Anhydrous sodium carbonate (Na_2_CO_3_) (Penta, Prague, Czech Republic). Ascorbic acid, 0.02 N I_2_ solution, and ammonium ferric citrate ((NH_4_)_5_[Fe(C_6_H_4_O_7_)_2_]) (Merck, Darmstadt, Germany). Homologous series of C8–C24 n-alkanes (Polyscience Corp., Niles, IL, USA). Citric acid (Acros Organics, Morris Plains, NJ, USA). Starch (Riedel-de Haën, Seelze, Germany). Potassium metabisulfite (K_2_S_2_O_5_) (Syndesmos S.A., Athens, Greece).

### 4.2. Raw Materials, Yeast, and Media

The FSS was obtained from the Agricultural Cooperatives’ Union of Aeghion S.A. (Aeghion, Greece). The composition of FSS and aqueous FSS extracts was provided in detail in a previous study [2].

The cryotolerant, alcohol-resistant yeast *S. cerevisiae* AXAZ-1, isolated from a vineyard of the Achaia region (Ano Ziria; 38.31282, 21.94757) and available at the Department of Chemistry of the University of Patras (Greece), was used for the winemaking experiments. It was grown at 30 °C in sterile medium containing (per liter) 20 g glucose, 4 g yeast extract, 1 g (NH_4_)_2_SO_4_, 1 KH_2_PO_4_, and 5 g MgSO_4_∙7H_2_O, and was harvested by centrifugation at 5000 rpm for 10 min (Sigma 3K12, Bioblock Scientific, Sigma Larborzentrifugen GmbH, Osterode, Germany) [2].

Alcohol for spirits production (potable) was obtained by the B.G. Spiliopoulos S.A. Distillery-Winery (Patras, Greece).

### 4.3. Preparation of FSS Extracts (Musts)

For winemaking, FSS was extracted by maceration with hot water (70 °C) to receive an extract with a hydrometer density of 15.5 °Be (Baumé) (264 g/L total sugar) [2]. The extract (must) was used for winemaking after K_2_S_2_O_5_ addition (to provide a stoichiometric equivalent of 40 mg SO_2_/L). The TTA of the must was also adjusted to 6.5 g tartaric acid/L by addition of tartaric acid.

### 4.4. Preparation of FSS Syrup

The FSS extract was also used for syrup production. Sulfite was added at a higher level in the extract (1.2 g SO_2_/L) to avoid spoilage and spontaneous fermentation until its further use. However, it was observed that this practice was not able to efficiently prevent spoilage. Therefore, after several experiments, sulfite addition in the extraction water instead of the final extract was found to be an effective strategy. Initially, the received FSS extract was centrifuged at 5000 rpm for 10 min (Sigma 3K12, Bioblock Scientific, Sigma Larborzentrifugen GmbH, Osterode, Germany) and the excess sulfite was oxidized to a residual concentration of 40 mg SO_2_/L by treatment with food-grade 30% H_2_O_2_ solution. Then, condensation by evaporation was carried out at 45 °C, under vacuum on a rotary evaporator (Heidolph WB2001, Schwabach, Germany), until a syrup of about 38 °Be density was obtained (~646 g/L total sugar). The condensation was carried out at low temperature, in order to avoid thermally induced reactions of sugars and deterioration in the syrup quality (loss of aroma, color, production of sugar degradation products) that might affect the quality of the produced wines [4]. The syrups were stored in closed containers in a dark place and at room temperature until further use.

### 4.5. Sweet Wine Making from FSS

#### 4.5.1. Production of Sweet Wine with Addition of FSS to Adjust Sweetness (SW-F Wine)

For SW-F wine making (Method 1, Figure 1), fermentation of 700 mL of FSSE (15.5 °Be) was carried out at 22 °C with 16.4 g/L (wet weight) of *S. cerevisiae* AXAZ-1 culture. The fermentation was monitored by measuring the density of the fermenting must versus time (Figure 2), until all sugar was utilized. Then, 250 g of FSS raisins was added per liter of wine and the whole was left for 6 days at 10 °C for sugar extraction, until a density of 6 °Be was obtained. The extracted FSS residues were then removed and the wine was stored at 0 °C for 1 week for stabilization. The produced wine was analyzed for pH, ethanol, methanol, sugar content, organic acids, sulfite content, TTA, VA, TPC, PPC, AC, and aroma volatile profile by GC-MS.

#### 4.5.2. Production of Sweet Wine with Addition of FSS Syrup to Adjust Sweetness (SW-S Wine)

In the same manner, SW-S wine making (Method 2, Figure 1) was carried out by fermentation of 700 mL of FSSE with *S. cerevisiae* AXAZ-1 at 22 °C. After the completion of fermentation (Figure 2), 100 mL of FSS syrup was added per liter of wine to obtain the desired sweetness (~6 °Be density). The wine was stored and analyzed as described above.

#### 4.5.3. Production of Sweet Wine with Potable Alcohol Addition (SW-A Wine)

For SW-A wine making (Method 3, Figure 1), the fermentation of FSSE took place under the same conditions as above, but when the density of the fermenting liquid reached ~6 °Be, potable ethanol was added to fix the alcoholic strength to 15% *v*/*v*. The fermentation in all cases was monitored by measuring the liquid density versus time (Figure 2). The wine was stored and analyzed as described above.

### 4.6. Analytical Methods

#### 4.6.1. Determination of Acidity

The pH was measured by a Cyberscan 10 pH-meter (Eutech Inst., Singapore). TTA (as g tartaric acid/L) and VA (after steam distillation; as g acetic acid/L) were determined by titration of 10 and 50 mL sample, respectively, with std 0.1 M NaOH solution.

#### 4.6.2. Determination of Ethanol and Methanol

Ethanol and methanol were determined on a Shimadzu GC-8A instrument carrying a Teknokroma column (100–130 °C; increased by 10 °C/min), flame ionisation detector (FID), and a C-R6A Chromatopack integrator. High-purity He with a flow of 20 mL/min (40 °C) was used as carrier gas. The combustion gas in the detector was a mixture of hydrogen and air at pressures of 0.6 and 0.2 kg/cm^2^, respectively. The injection port and FID temperatures were both 210 °C. A solution of 1% *v*/*v* 2-propanol was used as internal standard (IS). The samples were diluted as follows: 750 μL of sample and 500 μL of IS were mixed in a 25 mL volumetric flask and the volume was fixed with water. The injection volume was 2 μL, and determinations were based on standard curves.

Ethanol was also determined by distillation and determination of the specific gravity of the distillate using a Gay-Lussac alcoholmeter. The % vol. alcohol content was obtained after temperature corrections, using suitable conversion tables [23]. The results are presented as average values plus standard deviations.

#### 4.6.3. HPLC Analysis of Sugars and Organic Acids

Sugars (fructose, glucose, sucrose) were analyzed on a Shimadzu LC-9A HPLC instrument carrying a Nucleogel Ion 300 OA column, a CTO-10A column oven (set at 33 °C), a LC-9A pump, a RID-6A refractive index detector, and a DGU-2A degassing unit. The mobile phase was aqueous 0.017 M H_2_SO_4_ solution at a flow rate of 0.55 mL/min, and 1% *v*/*v* 2-propanol solution was used as IS. The samples were diluted as follows: 40 mL of sample and 500 μL of IS were mixed in a 25 mL volumetric flask and the volume was fixed with water. The samples were filtered through 0.2 μm syringe filters (Filtropur S 0.2, Sarstedt, Nümbrecht, Germany). The injection volume was 40 μL.

Organic acids (citric, tartaric, malic, succinic, acetic) were analyzed on a LC-2000 Series HPLC system (Jasco Inc., Kyoto, Japan) equipped with a size-exclusion column (Rezex ROA-Organic acid H+ (8%), 300 mm × 7.8 mm i.d., 8 μm particle size; Phenomenex), CO-2060 PLUS column oven (set at 22 °C), PU-2089 pump, AS 2050 PLUS autosampler, MD-2018 photodiode array detector (210 nm), and ChromNav software [2]. Isocratic separation was performed with 0.005 M H_2_SO_4_ as mobile phase (0.5 mL/min). The samples were filtered through 0.2 μm syringe filters and the injection volume was 10 μL. All chromatographic determinations were based on standard curves of sugars and organic acids.

#### 4.6.4. Determination of TPC, AC, and PPC

TPC and AC were determined by the Folin–Ciocalteu reagent method and the DPPH radical scavenging method, respectively, as described in detail in [2,4]. Specifically, 0.1 mL sample, 5 mL water, and 1 mL Folin–Ciocalteu reagent were added in 10 mL flasks and left for 30 min in the dark. Then 1 mL of 7.5% *w*/*v* Na_2_CO_3_ solution was added, the volume was fixed to 10 mL, and the mixture was left again for another 30 min in the dark. The absorbance was then measured at 725 nm (Jasco V-630 UV-vis spectrophotometer), versus a blank determination. The TPC was expressed as mg gallic acid (GA)/L of wine, with the aid of GA standard curves.

For AC determination, 3 mL of 137.6 μM methanolic DPPH solution and various amounts of sample (in the range 0.05–1 mL) were added in test tubes, and the volumes were fixed with methanol to 4 mL. The samples were left for 30 min in the dark and the absorbance was measured at 517 nm, against aqueous methanol solution as blank [2]. The results were expressed as mg ascorbic acid equivalents (mg AA/L) with the aid of standard AA curves.

Additionally, a spectrophotometric estimation of the PPC was carried out, based on the reaction with ferric ammonium citrate (EBC Method 9.11) [24]. Specifically, 20 mL sample, 15 mL carboxymethyl cellulose (CMC) solution (containing 10 g CMC and 2 g EDTA in 1 l water), 1 mL 3.5% ferric ammonium citrate solution (containing 16% Fe and prepared right before use), and 1 mL NH_3_ solution (1:2 in water) were added under stirring in a 50 mL volumetric flask. The volume was fixed to 50 mL with deionized water. The mixture was left for 10 min and the absorbance was measured at 600 nm (Jasco V-630 UV-vis spectrophotometer). At the same time a blank solution was prepared without the addition of NH_3_ (EBC, 1987). The PPC (mg/L) was calculated as the difference in absorbance between sample and blank multiplied by 820.

#### 4.6.5. Sulfite Analysis

For the determination of sulfite in the wines, a titrimetric method was applied as described in detail in [4], based on releasing bound sulfite as KHSO_3_ after treatment with KOH, followed by H_2_SO_4_ treatment to convert KHSO_3_ to H_2_SO_3_*,* and titration with 0.02 N iodine solution with starch indicator.

#### 4.6.6. Volatile Profile

The profile of volatile compounds was analyzed by headspace, solid phase micro-extraction gas chromatography–mass spectrometry (SPME GC-MS), as described in [3] with slight modifications. In brief, for the SPME sampling, 2 mL of wine sample, 7.5 mL water, 1 g NH_4_)_2_SO_4_, 500 µL of 1,4-dioxane (1000 mg/L; as IS) were mixed in a glass vial, sealed, and heated for 5 min in a water bath at 40 °C. The SPME fiber (DVB/CAR/PDMS, 2 cm; Sigma-Aldrich, Darmstadt, Germany) was then exposed to the headspace for 30 min. The GC-MS analysis took place on a GC-MS-QP2010 Ultra (Shimadzu Inc., Kyoto, Japan) instrument at the following conditions: exposure of SPME fiber in the injection port (240 °C, 5 min, split ratio 1/10); GC separation with He as carrier gas (36 cm/s) in a DB-Wax capillary column (30 m, 0.25 mm i.d., 0.25 μm film thickness, Agilent Technologies Inc., Santa Clara, CA, USA), with temperature rising from 40 °C (5 min), to 180 °C (by 5 °C/min), to 240 °C (by 30 °C/min; held 5 min); MS analysis by electron ionization (70 eV, 40–300 *m*/*z* mass scan range, source and interface set at 200 and 240 °C, respectively). Identification and semi-quantification (normalized peak areas %) were performed with the GC-MS Solution (ver. 4.30; Shimadzu), AMDIS (ver. 2.72; NIST), and NIST MS Search (ver. 2.2; NIST) software, and were based on comparison of: (i) retention indices (RI) of C8-C24 n-alkanes, authentic compounds and those available in NIST14 library (NIST, USA), (ii) MS data with those of reference compounds and those obtained from NIST14. The reliability of identification (RID) was considered at levels: A, agreement of RI and MS spectra with those of an authentic compound; B, agreement of RI (ΔRI < 20), and MS similarity match > 900; and C, at least ΔRI < 20 or MS similarity match > 800.

#### 4.6.7. Sensory Evaluation

Samples of sweet wines were examined for their sensory characteristics by 10 laboratory members. The testers were unaware of the type of each sample, and were not all trained in food tasting; therefore, the control can be considered preliminary and consumer-oriented [25]. The samples were coded randomly by three-digit numbers and were served to the testers at equal amounts at room temperature (~22 °C). The panel was specifically asked to evaluate each product in terms of clarity (clear, cloudy), color intensity (pale, medium, deep), color description, presence of sediment, aroma description, and intensity (light, medium, deep), sweetness (semi-sweet, sweet, very sweet), acidity (low, medium, high), alcohol (light, medium, strong), tannin (low, medium, high), body (light, medium, rich), taste description and intensity (low, medium, high), and aftertaste (sort, medium, long). A spider web diagram was also plotted based on the descriptive sensory evaluation scores (0–9 scale) for the above visual, olfactory, and gustative descriptors of the sweet wines.

#### 4.6.8. Statistical Analysis and Software

Significant differences between means of various data groups were checked by One-Way Anova or *t*-test (two populations), at the 0.05 level of significance, using the Microcal™ Origin^®^ software, version 6.0 (Microcal Software, Inc., Northampton, MA, USA).

## 5. Conclusions

In the frame of efforts to add value to the Mediterranean currant cultivation and processing sectors, which is essential for their sustainability, non-fortified sweet wine production is proposed from the industrial side-stream (FSS) of premium quality Corinthian currant processing. The methods involve complete fermentation using an alcohol-tolerant yeast to obtain at least 15% ethanol, followed by the addition of FSS or FSS syrup to adjust the desired sweetness, post-fermentation. The non-fortified wines had better oenological or nutritional characteristics, in terms of VA, AC, and TPC, and consumer preference, compared to the fortified wine. The syrup added to adjust sweetness could also be produced as a separate added value product alongside the sweet wine in the same industrial unit [4]. With these methods, it is possible to avoid fortification by additional alcohol, which is expensive due to high taxation and is usually made from molasses and may alter the typical aromas of grapes, raisins, and wine fermentation. The sweetness adjustment by FSS is the simplest and lowest cost method since it does not involve ethanol or syrup addition. In all cases, the proposed methods can lead to good quality sweet wines with a characteristic fruity (grape/raisin) flavor that could be commercialized as specialty raisin wines or beverages, thus adding value to a unique agricultural product of the Mediterranean area. Finally, since this study proposes efficient “sweet raisin wine” production methods, any disadvantages may be considered in terms of comparison with conventional winemaking, such as the additional aqueous extraction step, and the need for proper handling of the extracts to avoid spoilage (sulfite addition).

## Figures and Tables

**Table 1:** Composition of the sweet wines made from Corinthian currants finishing side-stream (FSS).

Parameter	SW-F	SW-S	SW-A
Total titratable acidity (g tartaric acid/L)	7.39 ± 0.16 ^a^	7.35 ± 0.13 ^a^	6.38 ± 0.25 ^b^
Volatile acidity (g acetic acid/L)	0.32 ± 0.01 ^a^	0.27 ± 0.03 ^b^	0.67 ± 0.09 ^c^
pH	3.88 ± 0.02 ^a^	3.86 ± 0.02 ^a^	3.83 ± 0.02 ^b^
Ethanol (% *v*/*v*)	15.3 ± 0.1 ^a^	15.0 ± 0.5 ^a^	16.0 ± 0.4 ^b^
Total sugars (g/L)	121.7 ± 6.9 ^a^	138.6 ± 5.9 ^b^	110.7 ± 5.7 ^c^
Glucose (g/L)	59.2 ± 3.8 ^a^	67.4 ± 3.0 ^b^	44.2 ± 4.0 ^c^
Fructose (g/L)	62.5 ± 3.1 ^a^	71.2 ± 2.1 ^b^	66.5 ± 1.7 ^c^
Saccharose (g/L)	nd	nd	nd
Free sulfite (mg/L)	14.9 ± 3.2 ^a^	11.9 ± 0.8 ^a^	11.5 ± 1.0 ^a^
Total sulfite (mg/L)	24.3 ± 4.6 ^a^	29.4 ± 2.6 ^a^	44.8 ± 4.5 ^b^
Citric acid (g/L)	0.66 ± 0.01 ^a^	0.86 ± 0.15 ^b^	0.55 ± 0.09 ^c^
Tartaric acid (g/L)	2.19 ± 0.07 ^a^	2.58 ± 0.20 ^b^	1.99 ± 0.15 ^a^
Malic acid (g/L)	3.26 ± 0.05 ^a^	3.62 ± 0.04 ^b^	3.49 ± 0.07 ^c^
Succinic acid (g/L)	2.84 ± 0.03 ^a^	2.84 ± 0.09 ^a^	1.98 ± 0.12 ^b^
Acetic acid (g/L)	0.46 ± 0.31 ^a^	0.31 ± 0.44	0.48 ± 0.23
Total phenolic content (mg GA/L)	325 ± 5 ^a^	284 ± 4 ^b^	244 ± 5 ^c^
Polyphenols (mg/L)	576 ± 5 ^a^	555 ± 5 ^b^	459 ± 5 ^c^
Antioxidant capacity (mg AA/L)	35.0 ± 0.6 ^a^	24.0 ± 0.2 ^b^	18.0 ± 0.4 ^c^

SW-F: Sweet wine with added FSS raisins to adjust sweetness. SW-S: Sweet wine with added FSS syrup to adjust sweetness. SW-A: Sweet wine with alcohol added to cease fermentation at the desired sweetness level. GA: Gallic acid. AA: Ascorbic acid. nd: not detected. Superscript letters in a row indicate statistical differences between treatments (*p* < 0.05). All assays were carried out at least in triplicate (n = 3–6).

**Table 2:** Volatiles identified by SPME GC-MS analysis (normalized peak areas %) in the headspace of sweet wines produced from Corinthian currants finishing side-stream.

Compound	RID	RI_ref_	RI	SW-F	SW-S	SW-A
Esters						
Methyl acetate	A	828	820.3	0.02	<0.01	<0.01
Ethyl acetate	A	888	882.3	10.48	8.73	5.03
Ethyl propanoate	A	953	949	0.08	0.05	0.04
Ethyl 2-methylpropanoate (ethyl isobutyrate)	A	961	958.2	0.08	0.06	0.04
Propyl acetate	A	973	967.8	<0.01	<0.01	0.01
2-Methylpropyl acetate (isobutyl acetate)	A	1012	1012	0.13	0.08	0.13
Ethyl butanoate (ethyl butyrate)	A	1035	1034.7	0.51	0.24	0.43
Ethyl 2-methylbutanoate (ethyl 2-methylbutyrate)	B	1051	1050.5	0.06	0.06	0.01
Ethyl 3-methylbutanoate (ethyl isovalerate)	B	1068	1066.1	0.03	0.03	<0.01
Butyl acetate	A	1074	1069.7	<0.01	<0.01	<0.01
3-Methylbutyl acetate (isoamyl acetate)	A	1122	1120	8.58	6.07	16.31
Ethyl pentanoate (ethyl valerate)	A	1134	1132.5	0.19	0.06	0.05
2-Methylpropyl butanoate (isobutyl butyrate)	B	1158	1157.1	<0.01	<0.01	<0.01
Ethyl (*E*)-2-butenoate	B	1160	1159.7	0.01	0.01	0.01
Pentyl acetate (amyl acetate)	B	1176	1171.2	<0.01	<0.01	0.01
3-Methylbutyl propanoate (isoamyl propanoate)	B	1185	1187.2	0.02	0.02	0.01
Butyl butanoate (butyl butyrate)	B	1220	1216.4	<0.01	<0.01	<0.01
Ethyl hexanoate (ethyl caproate)	A	1233	1231.5	10.07	7.35	15.51
3-Methylbutyl butanoate (isoamyl butyrate)	B	1259	1264.9	<0.01	0.01	0.02
Hexyl acetate	A	1272	1271.8	<0.01	<0.01	0.20
Ethyl 5-hexenoate	C	1271	1277.3	<0.01	<0.01	<0.01
Ethyl 3-hexenoate	C	1290	1292.5	0.01	0.01	<0.01
Ethyl heptanoate (ethyl capronate)	B	1331	1333.5	0.51	0.38	0.34
Ethyl 2-hydroxypropanoate (ethyl lactate)	A	1347	1343.7	0.03	0.03	0.01
Heptyl acetate	C	1377	1373.8	<0.01	<0.01	<0.01
Ethyl (*E*)-4-heptenoate	C	1380	1374	0.01	0.03	0.05
Ethyl octanoate (ethyl caprylate)	A	1435	1434.7	10.66	14.84	23.56
Ethyl 7-octenoate	C	1478	1486.4	0.14	0.08	0.06
Ethyl nonanoate (ethyl pelargonate)	A	1531	1537.8	0.10	0.05	0.05
Ethyl 2-hydroxy-4-methylpentanoate	C	1547	1545.7	0.03	0.02	0.01
Ethyl decanoate (ethyl caprate)	A	1638	1639.9	0.67	3.49	6.04
3-Methylbutyl octanoate (isoamyl octanoate)	B	1658	1661.7	0.02	0.04	0.04
Diethyl butanedioate (Diethyl succinate)	A	1680	1678	0.33	0.30	0.13
Myrtenyl acetate (2-pinen-10-yl acetate)	C	1698	1688.1	<0.01	<0.01	<0.01
Ethyl 9-decenoate	B	1694	1692.5	0.26	0.49	1.19
Ethyl 2-phenylacetate (ethyl benzeneacetate)	C	1783	1781.9	0.04	0.05	0.01
2-Phenylethyl acetate	A	1813	1811.2	0.27	0.43	1.28
Ethyl dodecanoate (ethyl laurate)	A	1841	1844.5	0.06	0.40	1.25
2-Phenylethyl propanoate	C	-	1880.4	<0.01	<0.01	<0.01
Ethyl 3-phenylpropanoate (ethyl dihydrocinnamate)	C	1893	1884	0.03	0.04	0.04
2-Phenylethyl butanoate (phenethyl butyrate)	B	1958	1964.6	<0.01	<0.01	0.01
Ethyl 3-methylbutyl butanedioate (Ethyl isopentyl succinate)	B	1901	1904.7	0.03	0.04	0.01
Octyl octanoate	B	2009	2014.5	0.21	0.13	0.15
Total				<43.67	<43.61	<72.06
Alcohols						
1-Propanol	A	1036	1042.6	0.06	0.05	0.03
2-Methyl-1-propanol (isobutanol)	A	1092	1097.6	1.90	1.77	0.65
1-Butanol	A	1142	1149.5	0.06	0.08	<0.01
1-Penten-3-ol (ethyl vinyl carbinol)	B	1159	1165.9	<0.01	<0.01	<0.01
3-Methyl-1-butanol (isoamyl alcohol)	A	1209	1212.8	37.23	36.77	16.88
1-Pentanol	A	1250	1255	0.01	0.01	0.01
4-Methyl-1-pentanol (isohexyl alcohol)	A	1314	1318.7	0.04	0.04	0.02
(*Z*)-2-Penten-1-ol	B	1318	1323.7	<0.01	<0.01	<0.01
3-Methyl-1-pentanol	B	1325	1331.4	0.05	0.05	0.03
1-Hexanol	A	1355	1357.7	0.27	0.24	0.10
(*Z*)-3-Hexen-1-ol	B	1382	1386.8	<0.01	<0.01	<0.01
3-Octanol	B	1393	1398.8	<0.01	<0.01	<0.01
(*E*)-2-Hexen-1-ol	B	1405	1409.2	<0.01	<0.01	<0.01
2-Octanol	A	1412	1425.5	0.03	0.03	0.02
1-Octen-3-ol	A	1450	1454.2	0.47	0.23	0.16
1-Heptanol	B	1453	1460	0.41	0.35	0.38
2-Ethyl-1-hexanol	A	1491	1493.5	0.06	0.08	0.02
(*E*)-2-Hepten-1-ol	C	1517	1514.2	<0.01	<0.01	<0.01
2-Nonanol	C	1521	1525.1	0.03	0.04	0.01
2,3-Butanediol isomer 1	C	1543	1544.3	0.06	0.09	0.03
1-Octanol	A	1557	1562.5	0.48	0.43	0.18
2,3-Butanediol isomer 2	C	1556	1581.4	0.02	0.04	0.01
(*E*)-2-Octen-1-ol	C	1614	1616.6	0.07	0.02	0.02
2-Furanmethanol (Furfuryl alcohol)	B	1660	1661.3	<0.01	<0.01	<0.01
1-Nonanol	B	1660	1664.9	0.22	0.27	0.09
3-(Methylthio)-1-propanol (methionol)	B	1719	1718.3	<0.01	<0.01	<0.01
2-Dodecanol	C	1813	1821.8	0.01	0.01	<0.01
Phenylmethanol (benzyl alcohol)	B	1870	1875.4	<0.01	0.01	<0.01
2-Phenylethanol (phenylethyl alcohol)	A	1906	1912.2	8.41	9.92	5.45
1-Dodecanol (lauryl alcohol)	B	1966	1972.4	0.46	0.24	0.13
1-Tetradecanol (myristyl alcohol)	C	2165	2181.5	0.33	0.05	0.04
Total				<50.70	<50.83	<24.25
Organic acids						
Acetic acid	A	1449	1448.6	0.51	0.84	0.31
Propanoic acid	B	1535	1538.2	<0.01	<0.01	<0.01
2-Methylpropanoic acid (isobutyric acid)	C	1570	1569.2	0.02	0.02	<0.01
Butanoic acid	B	1625	1628.5	<0.01	<0.01	<0.01
3-Methylbutanoic acid (isovaleric acid)	B	1666	1671	0.03	0.03	0.01
2-Methylbutanoic acid	C	1662	1672.2	0.01	0.02	0.01
Pentanoic acid (valeric acid)	B	1733	1737.4	<0.01	<0.01	<0.01
Hexanoic acid (caproic acid)	A	1846	1844.6	0.16	0.16	0.19
3-Methylhexanoic acid	C	-	1955	<0.01	<0.01	<0.01
Octanoic acid (caprylic acid)	A	2060	2062.5	0.45	0.69	1.16
Nonanoic acid	C	2171	2174.3	0.01	<0.01	0.01
*n*-Decanoic acid (capric acid)	Β	2276	2250.8	0.05	0.31	0.59
Total				<1.25	<2.07	<2.28
Carbonyl compounds						
Acetaldehyde	A	702	698.3	0.27	0.26	0.22
2-Methylpropanal (isobutyraldehyde)	B	819	807.9	<0.01	<0.01	<0.01
Butanal (butyraldehyde)	B	877	867.3	<0.01	<0.01	<0.01
2-Butanone (methyl ethyl ketone)	B	907	899.3	<0.01	<0.01	<0.01
2-Methylbutanal	B	914	908.1	0.02	0.01	<0.01
3-Methylbutanal (isovaleraldehyde)	B	918	911.4	0.13	0.07	0.02
2,3-Butanedione (Diacetyl)	A	979	968.3	0.03	0.02	<0.01
Hexanal	A	1083	1076.1	0.69	0.31	0.09
2-Heptanone	B	1182	1177.7	0.01	<0.01	<0.01
Heptanal (oenanthic aldehyde)	B	1184	1179	0.06	0.04	0.01
4-Methyl-2-heptanone	B	1206	1203.4	<0.01	<0.01	<0.01
3-Octanone (ethyl amyl ketone)	B	1253	1251.7	0.03	0.0	0.01
3-Hydroxy-2-butanone (acetoin)	A	1284	1280.6	0.03	0.01	<0.01
Octanal	B	1289	1284.2	0.49	0.51	0.08
2-Heptenal	B	1323	1318.6	0.04	0.04	0.01
6-Methyl-5-hepten-2-one	C	1338	1334.8	0.11	0.08	0.06
2-Nonanone	C	1390	1387.7	0.09	0.06	0.01
Nonanal	B	1391	1390.5	0.20	0.09	0.05
3-Octen-2-one	C	1411	1404.5	0.04	<0.01	<0.01
(*E*)-2-Octenal	C	1429	1425.8	0.08	0.12	0.07
2-Furfuraldehyde (furfural)	A	1461	1459.2	0.51	0.51	0.14
Decanal	B	1498	1498.2	0.01	0.01	<0.01
Phenylmethanal (benzaldehyde)	A	1520	1516.8	0.70	0.50	0.04
(*E*)-2-Nonenal	C	1534	1533.4	<0.01	<0.01	<0.01
(3*E*,5*E*)-3,5-Octadien-2-one	C	1570	1568.8	<0.01	<0.01	<0.01
5-Methyl-2-furfural	B	1570	1569.5	<0.01	<0.01	<0.01
6-Methyl-3,5-heptadiene-2-one	B	1602	1591.1	<0.01	<0.01	<0.01
Ethyl-1H-pyrrole-2-carboxaldehyde	C	1610	1605.3	<0.01	0.01	0.01
Phenylacetaldehyde	C	1640	1636.2	<0.01	<0.01	<0.01
2,4-Nonadienal	C	1700	1699.7	<0.01	<0.01	<0.01
2,4-Decadienal	B	1797	1805.2	0.01	0.01	<0.01
1H-Pyrrole-2-carboxaldehyde (pyrrole aldehyde)	B	2030	2022.9	<0.01	<0.01	<0.01
Total				<3.55	<2.71	<0.82
Terpenes						
d-Limonene (1-methyl-4-prop-1-en-2-ylcyclohexene)	A	1200	1185.4	0.08	0.04	0.10
trans-Rose oxide [tetrahydro-4-methyl-2-(2-methylpropenyl)-2H-pyran]	C	1365	1351.4	<0.01	0.01	<0.01
Verbenyl ethyl ether (4-ethoxy-2,6,6-trimethyl-bicyclo[3.1.1]hept-2-ene)	C	1377	1372.5	<0.01	<0.01	<0.01
Thujone [(1*S*,4*S*,5*R*)-4-methyl-1-propan-2-ylbicyclo[3.1.0]hexan-3-one]	B	1430	1416.6	0.04	0.02	0.02
Linalool (3,7-dimethyl-1,6-octadien-3-ol)	A	1547	1550.9	0.03	0.03	0.01
Fenchol (1,3,3-trimethylbicyclo[2.2.1]heptan-2-ol)	B	1582	1585.6	<0.01	<0.01	<0.01
L-4-Terpineol (4-methyl-1-propan-2-ylcyclohex-3-en-1-ol)	B	1593	1604.1	<0.01	<0.01	<0.01
β-Cyclocitral (2,6,6-trimethylcyclohexene-1-carbaldehyde)	C	1611	1618.2	<0.01	<0.01	<0.01
α-Terpineol [2-(4-methyl-3-cyclohexen-1-yl)-2-propanol]	A	1697	1700.5	0.02	0.01	<0.01
l-Borneol (1,7,7-trimethyl-bicyclo[2.2.1]heptan-2-ol)	B	1702	1704.1	<0.01	<0.01	<0.01
β-Citronellol (3,7-dimethyl-6-octenol)	A	1765	1767.7	0.06	0.12	0.03
Nerol [(*Z*)-3,7-dimethyl-2,6-octadien-1-ol]	B	1797	1799.8	<0.01	<0.01	<0.01
β-Damascenone	C	1823	1817.9	0.02	0.03	0.02
Geraniol (trans-3,7-dimethyl-2,7-octadien-1-ol)	A	1847	1849.6	<0.01	<0.01	0.01
trans-Geranylacetone [(*E*)-6,10-dimethylundeca-5,9-dien-2-one]	C	1859	1853.9	0.02	0.02	0.01
trans-β-Ionone [(*E*)-4-(2,6,6-trimethyl-1-cyclohexen-1-yl)-3-buten-2-one]	C	1940	1940.7	<0.01	<0.01	<0.01
Epicubenol [1*S*,4*R*,4a*S*,8a*R*)-4,7-dimethyl-1-propan-2-yl-2,3,4,5,6,8a-hexahydro-1H-naphthalen-4a-ol]	C	2067	2070	<0.01	<0.01	<0.01
T-Muurolol [(1*S*,4*S*,4a*R*,8a*S*)-1,6-dimethyl-4-propan-2-yl-3,4,4a,7,8,8a-hexahydro-2H-naphthalen-1-ol]	C	2186	2194.1	<0.01	<0.01	<0.01
α-Cadinol [(1*R*,4*S*,4a*R*)-1,6-dimethyl-4-propan-2-yl-3,4,4a,7,8,8a-hexahydro-2H-naphthalen-1-ol]	C	2226	2228.4	<0.01	<0.01	<0.01
Total				<0.29	<0.28	<0.21
Lactones						
Dihydrofuran-2(3H)-one (γ-Butyrolactone)	B	1632	1622.2	0.04	0.03	0.01
5-Methyl-2(5H)-furanone (β-Angelica lactone)	C	1669	1673.6	<0.01	<0.01	<0.01
Dihydro-5-ethyl-2(3H)-furanone (γ-hexalactone)	C	1694	1698.2	<0.01	<0.01	<0.01
5-Ethyl-2(5H)-furanone (2-hexen-1,4-lactone)	C	1745	1753.1	<0.01	<0.01	<0.01
6-Propyl tetrahydro-2H-pyran-2-one (δ-Octalactone)	C	1976	1978.4	0.05	0.08	0.03
Dihydro-5-pentyl-2(3H)-furanone (γ-Nonalactone)	C	2024	2028.8	0.02	0.05	0.03
Total				<0.12	<0.16	<0.07
Other compounds						
Dimethyl sulfide	B	754	738.9	<0.01	<0.01	<0.01
1,1-Diethoxy ethane (Acetal)	B	892	890.8	0.25	0.24	0.26
2-Ethylfuran	B	950	942.6	<0.01	<0.01	<0.01
1,4-Dioxane (IS)			1055.5			
2-Pentylfuran	B	1231	1225.6	0.02	0.02	0.01
2-Acetylfuran	B	1499	1500.4	0.02	0.02	<0.01
Methyl eugenol (1,2-dimethoxy-4-prop-2-enylbenzene)	C	2013	2013.2	<0.01	<0.01	<0.01
Total				<0.29	<0.29	<0.27
Hydrocarbons (alkanes/alkenes)						
Hexane	A	600	600	<0.01	0.01	<0.01
Heptane	A	700	700	<0.01	<0.01	<0.01
Octane	A	800	800	<0.01	<0.01	<0.01
1-Octene	C	847	831.8	<0.01	<0.01	<0.01
2-Octene	C	864	848.4	<0.01	<0.01	<0.01
Nonane	A	900	900	<0.01	<0.01	<0.01
Decane	A	1000	1000	<0.01	<0.01	<0.01
Dodecane	A	1200	1200	<0.01	<0.01	<0.01
Tetradecane	A	1400	1400	<0.01	<0.01	<0.01
Hexadecane	A	1600	1600	<0.01	<0.01	<0.01
Naphthalene	C	1746	1734	0.01	<0.01	0.03
Total				<0.03	<0.01	<0.04

SW-F: Sweet wine with added FSS raisins to adjust sweetness. SW-S: Sweet wine with added FSS syrup to adjust sweetness. SW-A: Sweet wine with added alcohol to cease fermentation at the desired sweetness level. RI: Retention Index. RIref: Reference RIs, obtained from the NIST14 library (as experimental RI median values from various literature sources). RID: Reliability of identification. A: Agreement of RI and MS spectra with those of an authentic compound analyzed under identical conditions. B: Agreement of RI (ΔRI < 20) and MS (match > 900). C: At least ΔRI < 20 or MS similarity match > 800.

**Table 3:** Sensory evaluation of the sweet wines produced from Corinthian currants finishing side-stream (FSS).

Descriptor	Description/Average Score					
	SW-F		SW-S		SW-A	
Appearance						
Clarity	Clear	9	Clear	9	Clear	9
Color intensity	Medium	5	Deep	9	Medium	5
Color description	Brown amber		Brown amber		Brown amber	
Sediment	no	0	no	0	no	0
Nose						
Aroma intensity	Medium	6	Medium	5	Medium	6
Aroma description	Fruity, raisin, grape		Fruity, raisin, grape		Fruity, raisin, grape	
Palate						
Sweetness	Sweet	9	Sweet	9	Sweet	6
Acidity	Medium	6	Medium	5	Medium	5
Tannin	Medium	4	Medium	4	Low	2
Alcohol	Strong	6	Strong	6	Strong	6
Body	Medium	5	Medium	6	Medium	4
Taste intensity	High	8	High	8	Medium	5
Taste description	Sweet, raisin		Sweet, raisin		Sweet, light fruity	
Aftertaste	Medium	6	Medium	6	Medium	5

SW-F: Sweet wine with added FSS raisins to adjust sweetness. SW-S: Sweet wine with added FSS syrup to adjust sweetness. SW-A: Sweet wine with added alcohol to cease fermentation at the desired sweetness level.

## Data Availability

Not applicable.

## Sample Availability

Not available.
